# Unravelling sociodemographic inequities in household secondhand smoke exposure among non-smokers in Malaysia: A secondary analysis of National Health and Morbidity Survey (NHMS) 2019

**DOI:** 10.18332/tid/208714

**Published:** 2025-10-30

**Authors:** Kuang Hock Lim, Yoon Ling Cheong, Jia Hui Lim, Sumarni Mohd Ghazali, Kee Chee Cheong, Chien Huey Teh, Pei Pei Heng, Ali Aman Marine, Mohd Hazilas Mat Hashim, Hui Li Lim

**Affiliations:** 1 Independent Researcher, Selangor, Malaysia; 2 Institute for Medical Research, National Institutes of Health, Ministry of Health Malaysia, Shah Alam, Malaysia; 3 School of Pharmacy, Faculty of Health and Medical Sciences, Taylor's University, Subang Jaya, Malaysia; 4 Sector of Biostatistics and Data Repository, National Institutes of Health, Ministry of Health Malaysia, Shah Alam, Malaysia; 5 Institute for Public Health, National Institutes of Health, Ministry of Health Malaysia, Shah Alam, Malaysia; 6 Institute for Clinical Research, National Institutes of Health, Ministry of Health Malaysia, Shah Alam, Malaysia

**Keywords:** secondhand smoke, non-smoker, home, NHMS, Malaysia

## Abstract

**INTRODUCTION:**

Secondhand smoke (SHS) exposure remains a significant global public health issue. Identifying the factors contributing to SHS exposure is crucial for developing targeted, evidence-based interventions to mitigate its impact. This study examines the patterns and determinants of SHS exposure at home among non-smoking Malaysians.

**METHODS:**

Data were derived from the National Health and Morbidity Survey (NHMS) 2019, which employed a cross-sectional design and multistage sampling to gather a representative sample of Malaysians aged ≥15 years. Demographic data and information on SHS exposure at home within the past month, were collected via a structured questionnaire. Weighted data were analyzed using chi-square tests and multivariable logistic regression.

**RESULTS:**

Of the 1876 participants (representing an estimated 3.54 million individuals), 19.8% (95% CI: 18.5–21.1) reported SHS exposure at home. Several sociodemographic factors were significantly associated with SHS exposure. Logistic regression analysis revealed higher odds of exposure among females (adjusted odds ratio, AOR=1.85; 95% CI: 1.55–2.29), Malays (AOR=2.65; 95% CI: 1.86–3.78), Bumiputera Sabah (AOR=4.16; 95% CI: 2.72–6.37), and Bumiputera Sarawak (AOR=3.67; 95% CI: 2.32–5.80). Other significant factors included being aged ≤24 years (AOR=1.87; 95% CI: 1.26–2.78) and belonging to a low income group (quintile 1; AOR=1.42; 95% CI: 1.12–1.95). Interaction analysis also identified significant two-way interactions between sex and some sociodemographic independent variables.

**CONCLUSIONS:**

Approximately two in ten non-smoking Malaysians were exposed to SHS at home. These findings underscore the need for comprehensive tobacco control measures that raise awareness about the health risks of SHS exposure at home. Educational campaigns should focus on promoting smoke-free home environments, particularly among the high-risk groups identified in this study.

## INTRODUCTION

Secondhand smoke (SHS), which is the smoke produced by active smokers, continues to be a significant global health threat^1,2^. Numerous studies have established a clear link between SHS exposure – also referred to as tobacco smoke pollution (TSP) or passive smoking – and various health issues in non-smokers, including lung cancer, heart disease, and asthma in children^1-4^. Diseases associated with SHS are among the top risk factors for global mortality, contributing to around 1.3 million deaths and approximately 37 million disability-adjusted life years (DALYs) in 2019, with 11.2% of this burden affecting children aged <5 years. In the EU, 526000 DALYs were lost due to SHS exposure at home^4^. Furthermore, research has shown that SHS exposure can hinder neurodevelopment in young individuals and is closely linked to poor academic performance and neurocognitive function^5^. Additionally, exposure to SHS has been associated with respiratory issues, increased risk of atopy, immunoglobulin E-mediated allergies, various allergic conditions, sleep disturbances, and depressive symptoms^6-8^.

As a result, reducing SHS exposure is a key objective in Malaysia’s national tobacco control strategy^9^. The strategy aligns with Article 8 of the World Health Organization’s Framework Convention on Tobacco Control (FCTC), which Malaysia ratified in 2005 ^10^. The Control of Tobacco Products Regulations (CTPR) have been introduced and amended multiple times to create smoke-free areas in public spaces. Numerous locations have been designated non-smoking zones, including air-conditioned and non-air-conditioned restaurants, government offices, healthcare facilities, public transport, schools, and indoor stadiums^11^. The Ministry of Health Malaysia and state governments have partnered to establish non-smoking zones in specific areas, such as Melaka’s ‘Melaka Bebas Asap Rokok’^12^ initiative. However, households are not covered under the FCTC, meaning home smoking bans must be adopted voluntarily. As a result, despite the increasing prevalence of smoke-free regulations in public spaces, the house remains a significant source of SHS exposure, particularly for vulnerable children^13^.

In light of this, the Malaysian government, through the Ministry of Health, has undertaken several initiatives to reduce SHS exposure at home. These include encouraging multisectoral cooperation in educational campaigns on the health harms of SHS and incorporating the smoke-free element into community-based intervention programs. The Ministry of Health Malaysia launched the community intervention program ‘Komuniti sihat, Pembina negara Kospen’ (Healthy Community, building the nation)^14^ to promote healthy lifestyles among the community, incorporating the smoke-free element into the program. The Ministry of Women, Family, and Community Development has also launched a smoke-free initiative within its program to reduce SHS exposure at home.

Previous studies have revealed that SHS exposure at home is higher among individuals with lower socioeconomic status, women, and those with lower education level^15^. A study by Lim et al.^16^, using the Global Adult Tobacco Survey-Malaysia data conducted in 2011, found that 27.9% of non-smokers (approximately 4.21 million individuals) were exposed to SHS at home at least once a month. The study also reported that higher odds of SHS exposure at home were observed among women, younger individuals, Malays (AOR=2.39; 95% CI: 1.56–3.64), other Bumiputera ethnic groups, self-employed individuals, and those working in the private sector^16^ However, these data are over 10 years old. With the changing demographic structure of the population, the implementation of anti-smoking measures, and the introduction of new tobacco products, the landscape of smoking among the Malaysian population and exposure to SHS at home may have changed.

In Malaysia, where there are 4.8 million tobacco users, secondhand smoke (SHS) exposure at home continues to be a significant public health concern. According to the NHMS survey, 31.0% of adults reported being exposed to tobacco smoke at home, with a notably higher rate among men (37.3%) compared to women (24.3%), and those living in rural (40.3%) versus urban areas (28.3%)^17^. While specific data on non-smokers and children are unavailable, likely, a significant number are also exposed to SHS at home, given that only 40.9% of respondents have enforced complete smoking bans within their households. This low rate of smoking restrictions indicates that many non-smokers living with smokers are frequently exposed to secondhand smoke^18^.

Therefore, this study aims to address the gap by using more recent data to determine the prevalence and factors associated with SHS exposure among non-smoking adults at home. The goal is to provide stakeholders with up-to-date insights, enabling them to formulate more proactive policies and measures.

## METHODS

### Data source

We derived the data from the NHMS 2019 study. A detailed description of the NHMS 2019 can be found elsewhere^17^. The NHMS is a cross-sectional study of a nationally representative sample of smokers and non-smokers conducted in Malaysia. The target population of the NHMS 2019 consists of Malaysian tobacco users and non-users, aged ≥15 years. Multistage sampling was employed to select a representative sample from the target population. The first stage involved stratification by all the states in Malaysia, further categorized into urban and rural classifications within each state. A two-stage random sampling method was employed within each stratum, with enumeration blocks (EBs) serving as the primary sampling units (PSUs) and living quarters as the secondary sampling units (SSUs). An EB is a designated geographical area with defined boundaries established by the Department of Statistics, Malaysia, typically containing about 80 to 120 living quarters (LQs). EBs were selected from each stratum using a probability-proportional-to-size approach, followed by 12 living quarters from each chosen EB. All individuals aged ≥15 years residing in the selected EBs were eligible to participate in the study.

### Data collection

Data collection was conducted through face-to-face interviews with trained research assistants. Before the interviews, participants were informed about the study, and their participation was entirely voluntary. All information gathered was used exclusively for research purposes, with safeguards in place to ensure the anonymity and confidentiality of the data provided. Informed written consent was obtained from all respondents. For participants aged <18 years, their written consent and permission from their parents/guardians were obtained.

### Questionnaire

We conducted our analysis using secondary data from the National Health and Morbidity Survey (NHMS) 2019. The data had been collected using a validated questionnaire available in both Malay and English. This instrument captured information on participants’ sociodemographic characteristics (such as gender, age, marital status, education level, occupation, and total monthly household income), smoking behaviors, use of smokeless tobacco, and exposure to secondhand smoke (SHS).

The smoking status of respondents was assessed using the question: ‘Do you currently smoke tobacco daily or less than daily?’. Respondents who answered ‘Yes’ were classified as ‘current smokers’, while those who responded with ‘Not at all’ were categorized as ‘non-smokers’. Only non-smokers were included in the present analysis. Exposure to secondhand smoke (SHS) at home was evaluated among all respondents using the question: ‘How often does anyone smoke inside your home?; Would you say daily, weekly, monthly, less than monthly, or never?’. Those who answered ‘daily’, ‘weekly’, or ‘monthly’ were classified as being exposed to SHS at home.

Independent variables included gender, age group (15–24, 25–34, 35–44, 45–54, 55–64, ≥65 years), ethnicity (Malay, Chinese, Indian, Bumiputera Sabah, Bumiputera Sarawak, and others), income (quintile 1 [lowest] to quintile 5 [highest]), education level (no formal education, primary education, secondary education, and tertiary education), marital status (single, married, divorced/widowed), and occupation (government, private sector, self-employed, and others[retired, homemaker, student]).

### Data analysis

We incorporated sex, age, and other independent variables to construct sampling weights, thereby reducing bias in other coefficients. Unless stated otherwise, the results were weighted using these sampling weights. The calculation of sampling weights began with a household weight at the level of enumerated households within the sampled EBs. Scaled up using the data weight to create a national-level household weight.

We used descriptive statistics tailored for complex survey data to estimate the prevalence of secondhand smoke (SHS) exposure among non-smokers in Malaysia. The associations between SHS exposure and factors such as demographics were analyzed using the Rao-Scott χ^2^ test. The multivariable logistic model included independent variables with a p≤0.25 in the bivariate analysis. All possible two-way interactions between the independent variables were assessed when producing the final model. Two-way interaction analysis between the independent variables revealed significant interactions among gender, marital status, education level, and age groups. Therefore, we ran two multivariable logistic regression (MLR) models separately for male and female respondents. The fit of each model was examined using a classification table. The multistage sampling design of the NHMS 2019 survey was analyzed using complex sample survey procedures available in SPSS Version 26 ^19^. Data are presented with a 95% confidence interval either to test the main effect or the interaction effect in the analysis.

## RESULTS

A total of 8994 respondents participated in the study, yielding a response rate of 83.1%. The majority of non-smokers in the study were female (78.7%), aged ≥55 years (specific age groups: 55–64 years, 83.6%; and ≥65 years, 89.7%), with no formal education (84.3%), and had tertiary education (87.0%). The ethnic distribution included Chinese (86.3%) and Indian descent (8.5%), and approximately 1 in 10 was from the divorced group (Table 1).

**Table 1:** Sociodemographic characteristics of non-smoking Malaysians aged ≥15 years from the National Health and Morbidity Survey 2019, using a cross-sectional study design (N=8994)

*Characteristics*	*Estimated* *population*	*Sample* *n*	*Percentage* *%*
**Gender**			
Male	6980454	3042	38.7
Female	11075291	5952	61.3
**Age** (years)			
15–24	4423563	1578	24.5
25–44	7343979	3015	40.7
45–64	4493287	2968	24.9
≥65	1794916	1433	9.9
**Ethnicity**			
Malay	9174994	5788	50.8
Chinese	4109999	1213	22.8
Indian	1192740	622	6.6
Other	3578011	1391	19.8
**Marital status**			
Single	5965041	2212	33.0
Married	10712404	5750	59.3
Divorce	1378300	1032	7.7
**Education level**			
No formal education	912031	569	5.1
Primary	3443813	2073	19.2
Secondary	8734052	4210	48.6
Tertiary	4868757	2101	27.1
**Occupation**			
Government servant	1276398	863	7.1
Private sector	5729823	2137	31.7
Self-employed	2485573	1321	13.8
Other	8559621	4669	47.4
**Income level**			
Quintile 1	3224769	1855	19.2
Quintile 2	3262709	1640	19.4
Quintile 3	3373531	1615	20.1
Quintile 4	3102747	1473	18.5
Quintile 5	3826716	1782	22.8

Our study found that approximately 1 in 5 non-smokers was exposed to secondhand smoke (SHS) at home during the last 30 days (19.9%), representing approximately 4.2 million adults in Malaysia. All the independent variables investigated in this study were significantly associated with SHS exposure at home. The proportion of female exposure to SHS at home was almost 10% higher than that of males (23.5%; 95% CI: 21.9–25.3 vs 13.8%; 95% CI: 12.1–15.7). SHS exposure was more than twice as high among Malays, Bumiputera Sabah, and Bumiputera Sarawak compared to Chinese and Indian non-smokers. A significantly lower exposure to SHS at home was also observed among individuals working in the government sector, those with tertiary education, and those in the higher income quintile (quintile 5) (Table 2).

**Table 2:** Prevalence and factors associated with SHS exposure at home, among non-smoking Malaysians aged ≥15 years from the National Health and Morbidity Survey 2019, using a cross-sectional study design (N=8994)

*Variables*	*Estimated* *population*	*Sample* *n*	*Percentage* *%*	*95% CI*	p*
**Gender**					
Male	955207	399	13.8	12.1–15.7	<0.001
Female	2588783	1477	23.5	21.9–25.3	
**Age** (years)					
15–24	1130940	406	25.7	22.6–29.1	<0.001
25–44	1468101	694	20.1	18.2–22.2	
45–64	740131	564	16.6	14.8–18.6	
≥65	204817	212	11.6	9.5–14.0	
**Ethnicity**					
Malay	2058255	1324	22.6	21.2–24.1	<0.001
Chinese	347474	113	8.5	6.4–11.3	
Indian	94624	64	8.0	5.6–11.3	
Other	752319	375	29.3	25.6–33.3	
**Marital status**					
Single	1333933	497	22.5	20.1–25.2	0.002
Married	2009737	1200	18.9	17.4–20.5	
Divorce	200319	179	14.9	12.0–18.2	
**Education level**					
No formal education	177123	134	19.7	15.4–24.9	<0.001
Primary	666131	443	19.5	17.0–22.2	
Secondary	1978943	948	22.8	20.9–24.9	
Tertiary	706721	342	14.6	12.6–16.8	
**Occupation**					
Government servant	202909	121	16.0	12.4–20.5	0.001
Private sector	1018481	438	17.9	15.8–20.2	
Self-employed	425129	268	17.9	14.6–20.3	
Other	1896939	1048	22.3	20.4–22.4	
**Income level**					
Quintile 1	774567	438	24.2	21.2–27.5	<0.001
Quintile 2	715918	386	22.1	19.3–25.1	
Quintile 3	755377	367	22.5	19.5–25.8	
Quintile 4	620616	303	20.1	16.8–23.8	
Quintile 5	482389	277	12.8	10.8–15.1	

* Rao-Scott chi-squared analysis. Further details are provided in the Supplementary file.

**Figure 1:**
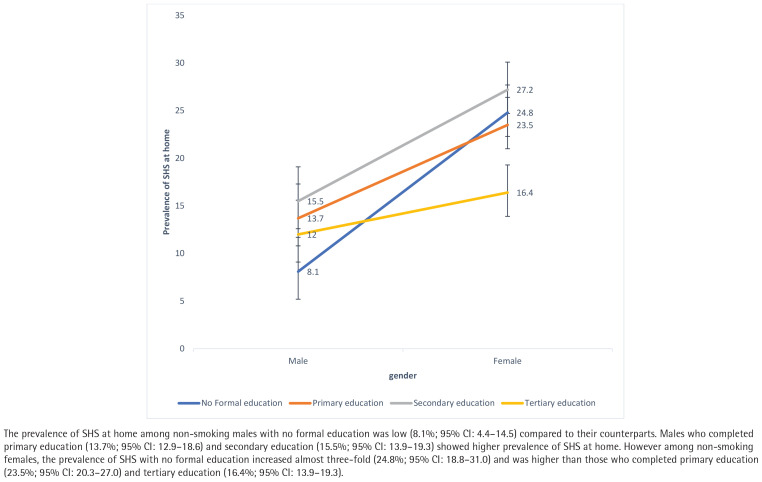
Interaction between gender and education level among non-smoking Malaysians who participated in the NHMS 2019 study

**Figure 2:**
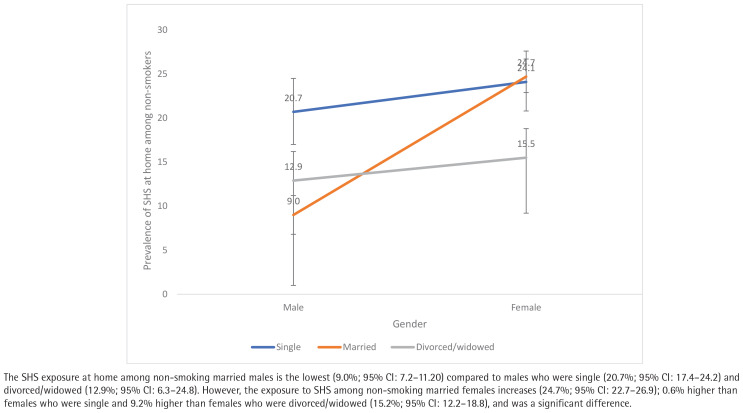
Interaction between gender and marital status among non-smoking Malaysians participating in the NHMS 2019 study

**Figure 3:**
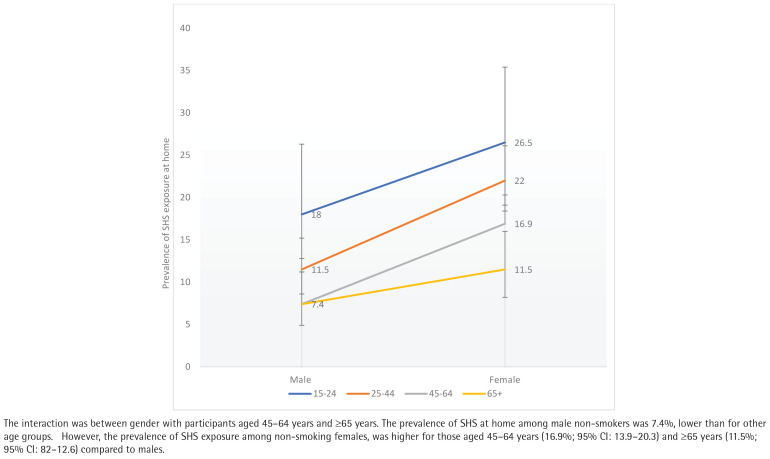
Interaction between gender and age groups among non-smoker Malaysians who participated in the NHMS 2019 study

Interaction analysis between the independent variables revealed a significant interaction between gender and education level (Figure 1), gender and marital status (Figure 2), and gender and age group (Figure 3). Therefore, a separate multivariable logistic regression was run for gender.

Table 3 shows that, after adjusting for confounding factors, the odds of SHS exposure at home were significantly higher among both non-smoking Malays (males, AOR=3.02; 95% CI: 1.93–5.95; females, AOR=2.24; 95% CI: 1.53–3.26) and other ethnicities (males, AOR=3.77; 95% CI: 1.86–7.64; females, AOR=3.24; 95% CI: 2.09–5.01) compared to non-smoking Chinese. In addition, females in younger age groups (15–24 years, AOR=1.76; 95% CI: 1.09–2.86; 25–44 years, AOR=1.61; 95% CI: 1.09–2.36) are more likely to be exposed to SHS at home (reference: ≥65 years) but no similar findings were reported among males. However, the type of occupation was not significant after adjusting for confounding variables.

**Table 3:** Multivariable logistic regression analysis of secondhand smoke exposure at home by sociodemographic variables, among non-smoking Malaysians aged ≥15 years from the National Health and Morbidity Survey 2019, using a cross-sectional study design (N=8259)

*Variables*	*Male* *(N=3278)*		*Female* *(N=4981)*	
	*AOR*	*95% CI*	*AOR*	*95% CI*
**Age (years)**				
15–24	1.65	0.81–3.35	1.76	1.09–2.86
25–44	1.30	0.66–2.58	1.61	1.09–2.36
45–64	0.91	0.42–1.97	1.35	0.95–1.90
≥65 ®	1		1	
**Ethnicity**				
Malay	3.02	1.53–5.95	2.24	1.53–3.26
Chinese ®	1		1	
Indian	0.91	0.33–2.50	0.60	0.34–1.06
Others	3.77	1.86–7.64	3.24	2.09–5.01
**Marital status**				
Single	1.90	1.21–2.99	0.94	0.68–1.29
Married ®	1		1	
Divorce	1.85	0.46–4.51	0.65	0.48–0.88
**Education level**				
No formal education	0.35	0.14–0.89	1.92	1.17–3.14
Primary	1.05	0.61–1.80	1.62	1.17–2.26
Secondary	1.05	0.70–1.58	1.85	1.44–2.39
Tertiary ®	1		1	
**Occupation**				
Government servant ®	1		1	
Private sector	1.46	0.80–2.66	0.95	0.61–1.42
Self-employed	1.13	0.57–2.22	1.02	0.64–1.62
Other	1.05	0.50–2.22	1.01	0.66–1.52
**Income level**				
Quintile 1	1.41	0.80–2.51	1.52	1.10–2.09
Quintile 2	1.40	0.84–2.35	1.32	0.98–1.83
Quintile 3	1.65	0.99–2.77	1.37	0.98–1.92
Quintile 4	1.38	0.84–2.25	1.32	0.93–1.88
Quintile 5 ®	1		1	
Classification table accuracy	86.0%		75.1%	

AOR: adjusted odds ratio. ® Reference categories

## DISCUSSION

This study is the second to describe Malaysian non-smokers exposed to secondhand smoke (SHS) at home after Lim et al.^16^ described it using GATS-M 2011 data. This study found that approximately 20% of non-smoking adults were still exposed to SHS at home. This prevalence is lower than Tripathy’s the findings of Tripathy^20^ among non-smokers in the Indian GATS 2016–2017 (29.2%). Similarly, the prevalence is lower than the 40.3% reported among adults in Saudi Arabia^21^ and 46.7% in China^22^ but approximately 6% higher than the 13.8% SHS exposure reported among adults in eight countries in Sub-Saharan Africa from 2012 to 2018^15^. It is also nearly eight times higher compared to non-smokers in Australia^23^ This finding may be due to differences in the tobacco control environment, enforcement levels, and smoking prevalence across these countries, with Australia being one of the countries enforcing strict anti-smoking legislation and having lower smoking prevalence. However, the prevalence of almost 20% is lower than the study by Lim et al.^16^ which reported 27.9%.

This decrease is consistent across all sociodemographic variables, which is very encouraging. A similar finding was reported by Verma et al.^24^ in their GATS II study in India, showing a decrease in SHS exposure at home from 48% to 35%. Furthermore, a similar trend was also reported by Huang et al.^25^, who noted that from 2010 to 2018, the percentage of indoor smokers decreased from 84.7% to 71.9% among respondents aged 15–64 years in China^25^. The decrease in SHS exposure observed in this study may be attributed to anti-tobacco policies introduced in Malaysia, such as KOSPEN by the Ministry of Health, Malaysia^14^, which was launched at the community level and includes smoke-free home elements, as well as initiatives by the Ministry of Women and Community Development. Although the 2004 tobacco control regulations do not ban smoking at home, smoking bans in public places may have created a social norm. The reduction in home SHS exposure over time can be attributed to a ‘norm-spreading’ effect and a shift toward reduced social acceptability. Additionally, the Malaysian government has launched several initiatives in response to challenges identified in previous studies on smoking practices, including investments in public awareness campaigns targeting diverse audiences. The government has also established public–private cooperation by incorporating tobacco cessation into the healthcare system and encouraging healthcare facilities to offer cessation services, which may have contributed to the findings of this study.

The study found that females had a higher prevalence of SHS exposure. The prevalence among females is almost identical to the findings by Yang et al.^26^ among women in 53 LMICs (23.0%; 95% CI: 22.8–23.2). Similar findings were reported by Lim et al.^16^ and several other researchers, including Verma et al.^24^ in the GATS II study in India (38.1% vs 30.7%, AOR=1.5) and in a study by Ngabose et al.^27^ in South Africa. The study also found that the odds of exposure were higher among married females. We postulate that this exposure is primarily due to spouses who smoke, which revealed that the exposure of females to SHS increased significantly compared to their counterparts who were single or widowed. These findings may be because individuals who smoke are often the heads of the family, and respect for the head of the family, combined with a desire to maintain family harmony, leads women to be less likely to protest against their husband’s smoking behavior at home^18^. Since women often have no alternative but to be at home and cannot avoid SHS exposure, in addition, they may not reprimand their spouses for their behavior and lack the authority to make the house a smoke-free area. This finding suggests that community-level interventions involving family heads must be improved and strengthened. The study found that perceptions of SHS risk encourage parents to restrict smoking in the home or car^27,28^. However, male respondents who were married were less likely to be exposed to SHS at home. The findings are contradicted by the findings of Lim et al.^16^. These findings are also incongruent with the ‘marriage protection’ and ‘marriage selection’ theories^29^, which suggest that emotional distress from divorce may lead divorcees to turn to smoke for relief. Previous findings indicated that married individuals tend to have more economic advantages and receive more social and psychological support^30^.

The study found that non-smoking Malay adults, as well as other ethnicities of both genders, are more likely to be exposed to SHS at home. This finding is consistent with the results reported by Lim et al.^16^ using GATS study data. The finding could be due to the high prevalence of smoking in these ethnic groups, which has remained unchanged for the past decade. Additionally, their tendency to create smoke-free homes is lower, making non-smoking adults in these groups more vulnerable to SHS exposure. As the U.S. Surgeon General’s report suggests, adopting voluntary smoke-free home rules is a strategy to protect non-smokers, such as children, from SHS exposure at home^6^.

There are no significant odds of exposure to SHS among males of different age groups in the study, which contradicts the findings by Lim et al.^16^ using the GATS 2011 data. This finding may be due to male adults being more mobile and often the primary breadwinners in the household; therefore, the time they spend at home is less compared to females, who typically spend most of their time at home. Thus, no difference in likelihood was observed in the study. However, there is a significant difference in the odds of SHS exposure at home. Our analysis revealed that females in the older group (≥65 years) are less likely to be exposed to SHS at home compared to their counterparts aged 15–24 and 25–44 years. The findings align with those of Lim et al.^16^ and other researchers, including Verma et al.^24^, who reported a significantly lower prevalence among non-smokers aged ≥45 years. The results may be due to the low prevalence of smoking among respondents aged ≥65 years in this study, reducing their likelihood of SHS exposure. However, the opposite trend was observed among respondents aged ≤24 years. Despite low smoking prevalence in this age group, the likelihood of SHS exposure is higher, possibly due to their lack of economic independence and living with family members, which increases their exposure risk. Another factor may be the cultural tradition in Malaysia of respecting elders, which discourages challenging elderly members’ smoking behavior at home. These findings are concerning because studies have shown that children or young people exposed to SHS at home are more likely to smoke in the future due to the normalization of smoking by significant others^6^.

The study showed that non-smoking females with tertiary education were less likely to be exposed to SHS at home compared to their counterparts with lower level of education. These findings contrasted with those of Lim et al.^16^ who found no significant association between education level and SHS exposure. However, this finding aligns with Verma et al.^24^ in India and Jin et al.^22^ in China, who reported consistent findings that individuals with lower level of education and literacy were more than twice as likely to be exposed to SHS at home compared to those with higher level of education. Higher education can increase awareness of the health risks associated with smoking, which may play a key role in reducing SHS exposure. For example, Cheah et al.^29^ found that people with lower level of education in Malaysia were less likely to recognize the dangers of passive smoking compared to those with higher level of education. Educated individuals, especially females, may have more knowledge about SHS exposure and take steps to reduce it. Second, women’s relatively higher level of education increases their bargaining power and ability to implement health-protective rules and norms, such as advising spouses to practice smoke-free homes and avoid SHS exposure^31^, as shown in previous studies.

Additionally, people with tertiary education are typically employed, which limits their time at home. In our study, 29.0% were in the Government sector, 29.9% in the private sector, and 9.3% were self-employed. However, we did not find a similar trend among non-smoking males. There was no significant association between the respondents with primary, secondary, and tertiary education and the risk of association with SHS exposure at home. And male respondents with no formal education are less likely to be exposed to SHS at home compared to others. Further analysis revealed that most of the males with non-formal education consisted of a substantial proportion aged ≥65 years (42.2%), which is a lower prevalence of smoking and showed a lower risk of exposure^8^. However, the findings require further investigation in future studies.

Non-smoking respondents among males and females who were government employees had a level of SHS exposure similar to those employed in the private sector or self-employed. This finding contrasts with the study of Lim et al.^16^, which reported higher SHS exposure among smokers in the private sector and self-employed groups. The difference may be due to a decrease in SHS prevalence of >10% among private sector and self-employed respondents from 2011 to 2019, potentially due to the expansion of smoke-free areas in the workplace, and the KOSPEN WOW intervention programs in the workplace might have contributed to the lower prevalence of smoking among respondents working in private sector or self-employed^32^. However, the findings also indicated that the smoke-free areas at all government facilities which Ministry of Health, Malaysia had implemented in the last two decades still cannot influence the government servant to reduce SHS exposure at home and contradict Bronfenbrenner ecology^33^ that the changes in any system will influence other systems, and findings by Lim et al.^34^ who reported that respondents working in smoke-free areas more likely to implemented a smoke-free home. Therefore, all stakeholders should carry out more intervention and health promotion activities to enhance this group’s needs.

In contrast with Lim et al.^16^, who found no association between SES and SHS exposure among male non-smokers and females from quintile two and quintile five. However, it is consistent with Nazar et al.^15^ report that 11 out of 15 LMIC countries showed a decrease in SHS exposure at home following an increase in SES. Similarly, studies in India^24^ and China^22^ have reported similar findings. The KOSPEN community program, developed by the Ministry of Health, may have contributed to these findings by promoting a healthy lifestyle and behavior modification among the community. However, the non-smoking female from quintile 1 reported higher odds of SHS exposure at home. The finding required that the community intervention program should focus more on this group and their spouse.

### Strengths and limitations

The sample chosen for this study was representative of the Malaysian population aged ≥15 years, allowing us to generalize the findings to the broader Malaysian population. Trained interviewers collected data using a standardized procedure, minimizing systematic bias. However, there are limitations. The cross-sectional design restricts the ability to draw causal conclusions. Additionally, SHS exposure was self-reported rather than measured objectively, making the data susceptible to recall bias. Several significant variables, such as smoking restrictions at home, knowledge, and attitudes toward SHS, were not investigated. The Institute of Public Health, National Institute of Health, Ministry of Health, Malaysia, collected this information six years ago, and it may be outdated. Additionally, the study did not examine the dimensions of SHS exposure, such as frequency and intensity. Despite these limitations, the study provides valuable insights into SHS exposure at home among non-smokers in Malaysia.

## CONCLUSIONS

The NHMS 2019 revealed that a significant proportion of Malaysians were exposed to SHS at home, although there was some reduction compared to 2011. This reduction is likely due to tobacco control and intervention measures implemented in Malaysia for the last eight years. Therefore, all stakeholders should enhance tobacco control measures, health promotion, and intervention programs should be improved, and focus on encouraging voluntary smoking bans in homes, with efforts to address social norms surrounding SHS exposure. Smoke-free policies have positively influenced social norms regarding SHS exposure at home.

## Supplementary Material



## Data Availability

The data supporting this research are available from the authors on reasonable request.
