# Supplementary material for: Unravelling sociodemographic inequities in household secondhand smoke exposure among non-smokers in Malaysia: A secondary analysis of National Health and Morbidity Survey (NHMS) 2019
Source: Tob Induc Dis. 2025 Oct 30;23:10.18332/tid/208714. doi: 10.18332/tid/208714 (PMC12574549; doi:10.18332/tid/208714)
Supplement: Supplementary file 1 [file TID-23-164-s1.pdf]

Supplementary file

Sociodemographic characteristics or parental factors	Sociodemographic characteristics or parental factors	Percentage (95% CI)
**Gender**	**Education Attainment**	
Male	No formal education	8.1(4.4-14.5)
Female	No formal education	24.4(18.8-31.0)
Male	Primary Education	13.7(10.1-18.2)
Female	Primary Education	23.5(20.3-27.0)
Male	Secondary Education	15.5(12.9-18.6)
Female	Secondary education	27.2(24.6-30.0)
Male	Tertiary Education	12.0(9.1-15.6)
Female	Tertiary Education	16.4(13.9-19.3)
**Gender**	**Marital Status**	
Male	Single	20.7(17.4-24.4)
Female	Single	24.1(20.6-27.9)
Male	Married	9.0(7.2-11.2)
Female	Married	24.7(22.7-26.9)
Male	Divorce/separated/widow	12.9(6.3-24.8)
Female	Divorce/separated/widow	15.2(12.2-18.8)
**Gender**	**Age group**	
Male	15-24	18.0(12.8-26.3)
Female	15-24	26.5(19.1-35.4)
Male	25-44	11.5(8.6-15.2)
Female	25-44	22.0(18.4-26.1)
M Male	45-64	7.4(4.8-11.2)
Female	45-64	16.9(13.9-20.3)
Male	65 and above	7.4(4.9-11.2)
Female	65 and above	11.5(8.2-16.0)

© 2025 Lim K.H. et al.
